# Agreement between pedometer and accelerometer in measuring physical activity in overweight and obese pregnant women

**DOI:** 10.1186/1471-2458-11-501

**Published:** 2011-06-27

**Authors:** Tarja I Kinnunen, Peter WG Tennant, Catherine McParlin, Lucilla Poston, Stephen C Robson, Ruth Bell

**Affiliations:** 1 Institute of Health and Society, Newcastle University, Newcastle upon Tyne, UK; 2 Institute of Cellular Medicine, Newcastle University, Newcastle upon Tyne, UK; 3 School of Health Sciences, University of Tampere, Tampere, Finland; 4 Newcastle upon Tyne Hospitals NHS Trust, Newcastle upon Tyne, UK; 5 Maternal and Fetal Research Unit, King's College, London, UK

## Abstract

**Background:**

Inexpensive, reliable objective methods are needed to measure physical activity (PA) in large scale trials. This study compared the number of pedometer step counts with accelerometer data in pregnant women in free-living conditions to assess agreement between these measures.

**Methods:**

Pregnant women (n = 58) with body mass index ≥25 kg/m^2 ^at median 13 weeks' gestation wore a GT1M Actigraph accelerometer and a Yamax Digi-Walker CW-701 pedometer for four consecutive days. The Spearman rank correlation coefficients were determined between pedometer step counts and various accelerometer measures of PA. Total agreement between accelerometer and pedometer step counts was evaluated by determining the 95% limits of agreement estimated using a regression-based method. Agreement between the monitors in categorising participants as active or inactive was assessed by determining Kappa.

**Results:**

Pedometer step counts correlated moderately (r = 0.36 to 0.54) with most accelerometer measures of PA. Overall step counts recorded by the pedometer and the accelerometer were not significantly different (medians 5961 vs. 5687 steps/day, p = 0.37). However, the 95% limits of agreement ranged from -2690 to 2656 steps/day for the mean step count value (6026 steps/day) and changed substantially over the range of values. Agreement between the monitors in categorising participants to active and inactive varied from moderate to good depending on the criteria adopted.

**Conclusions:**

Despite statistically significant correlations and similar median step counts, the overall agreement between pedometer and accelerometer step counts was poor and varied with activity level. Pedometer and accelerometer steps cannot be used interchangeably in overweight and obese pregnant women.

## Background

Current recommendations emphasize that regular moderate intensity leisure-time physical activity (PA) during an uncomplicated pregnancy may have benefits such as reducing fatigue, back pain, stress and depression and improving glycaemic control, but has no known harmful effects on the health of the mother or the fetus [1,2]. However, the available evidence is limited and larger and better quality trials are needed to define the potential role for PA promotion in preventing pregnancy complications such as gestational diabetes and pre-eclampsia [3].

Most previous studies assessing PA in relation to pregnancy outcome have used questionnaires or other self-reported measurements of PA, as these are cheap to administer in large scale studies [4]. Although some of the questionnaires have been validated in pregnant women either against accelerometer [5-7], a portable activity monitor [8] or pedometer and a PA log book [9], their validity has usually been low or moderate, especially with regard to the light intensity activity that is common among pregnant women [10]. These limitations are also observed for PA questionnaires in other populations [11,12]. Therefore, inexpensive, objective PA measurement methods are needed for large scale studies to obtain more accurate information on PA levels during pregnancy.

Accelerometers and pedometers are the most commonly used objective methods of assessing PA in epidemiologic studies, and have been used in a number of previous studies of pregnant women [5,6,10,13-16]. While accelerometers provide more detailed information on PA than pedometers, pedometers are much less expensive and therefore more economically feasible for larger studies [17]. It is unclear whether pedometers and accelerometers provide comparable estimates of PA in pregnant women. This issue was recently explored in two small studies (n = 30 in both of them) examining pregnant women in free-living conditions [18] and on a treadmill [19]. Similar comparisons have also been reported in healthy adults [20,21] adults with human immunodeficiency virus [22] and older people [23] in free-living conditions. These studies suggest that pedometer and accelerometer step counts are highly correlated, but large individual differences in step counts exist. Nevertheless, pedometer step counts for assessing overall PA were advocated in most of these studies [18,21-23].

This study was designed as a preliminary investigation to determine appropriate PA measurement methods for a large randomised controlled trial of a lifestyle intervention in obese pregnant women. Overweight and obese women have a higher risk of several pregnancy complications and may benefit from increasing their PA levels during pregnancy [24,25]. The aim of this study was to compare pedometer step counts with several accelerometer-derived measures of PA in overweight and obese pregnant women in free-living conditions.

## Methods

### Study participants

Participants were overweight and obese pregnant women with body mass index (BMI) at least 25 kg/m^2 ^based on self-reported height and measured weight at the first visit to antenatal care, usually before 12 weeks' gestation. The exclusion criteria were BMI less than 25 kg/m^2^, age less than 16 years, multiple pregnancy, abnormal ultrasound scan result, complicated medical problems, inadequate language skills in English, or inability to give written informed consent. A research midwife recruited the participants when they attended for their routine ultrasound scan at either 11-14 or ≥20 weeks' gestation at the Royal Victoria Infirmary, Newcastle upon Tyne, UK, between July and December 2009. The participants were recruited in early pregnancy because information on appropriate PA measurement methods for the intervention study starting in early pregnancy was needed.

A total of 286 women were eligible for the study and 93 (33%) agreed to participate. All participants signed a written informed consent for participation. Ethical approval for the study was obtained from St Thomas's Hospital Research Ethics Committee, London, UK (National Research Ethics Service, REC reference 09/H0802/5).

### Data collection

This study was a cross-sectional comparison of two objective PA measurement methods. The research midwife asked participants to wear an accelerometer and a pedometer for four consecutive days, including one weekend day. In adults, three to five days of monitoring by accelerometer usually provides a reliable estimate of PA [26]. The participants kept a diary to record when the monitors were put on and when they were taken off. Data on participants' demographic details were collected using a short structured questionnaire. An appointment for returning the monitors was arranged after the four-day period.

#### Accelerometer

The GT1M Actigraph accelerometer used in this study is a small uniaxial monitor, which detects vertical accelerations over a user-specific time interval (epochs) [27]. The former version of the Actigraph accelerometer (Computer Science and Applications (CSA) 7164 model) is one of the most extensively validated accelerometers and its activity counts correlate reasonably with doubly labelled water derived energy expenditure in non-pregnant populations [28].

Participants were asked to wear the accelerometer on the right hip during waking hours except while swimming or having a shower or bath. They were given the choice of belt or waistband attachment and information was recorded on which they found to be the most comfortable. Most participants (n = 40, 69%) wore the accelerometer using a belt, while 14 (24%) clipped it on the waistband of their clothing and the status was unknown for four participants (7%). A 60-second epoch length was used in this study. The raw data was processed using the MAHuffe program (http://www.mrc-epid.cam.ac.uk/Research/Programmes/Programme_5/InDepth/Programme%205_Downloads.html). Periods of at least 60 min with no counts and days with less than 500 min of total valid recording time were excluded.

The following cut points were used to assess time spent at different intensity levels: sedentary activity <100 counts/min [29], light activity 100-1951 counts/min, moderate activity 1952-5724 counts/min and vigorous activity >5724 counts/min [30]. These cut points were originally developed for CSA Model 7164 accelerometer. Currently there is no consensus on the best cut points to be used and these may vary in different populations. The Freedson cut points, derived from treadmill conditions, were selected because leisure time PA in our population mainly consisted of walking and because these cut points have been used in previous studies comparing pedometers to accelerometers [18,20-22].

#### Pedometer

The Yamax Digi-Walker CW-701 (Yamax, Corp) was used to measure daily step counts. Yamax Digi-Walker models have been shown to be among the most accurate models in measuring step counts [31,32]. The participants were asked to wear this device during the same time period as the accelerometer. The participants clipped the pedometer either to the accelerometer belt or to the waistband of their clothing depending on how the accelerometer was attached.

### Categorising participants as active or inactive

Three different criteria were used to categorise participants as active: 1) ≥30 min MVPA/day (for accelerometer data only as this information could not be derived from the pedometer data), 2) ≥10,000 steps/day and 3) ≥8,000 steps/day. The first criterion was based on current PA recommendations [1,2]. The second criterion is a commonly used step target in health promotion and has been shown to be associated with health benefits [33,34]. The third criterion was chosen as there is some evidence to suggest that 8000 steps/day corresponds to 30 min of MVPA/day measured by accelerometer when using similar intensity cut points as in the present study [20,21,34].

### Statistical analyses

All activity data was averaged over the valid days of recording. The majority of variables were not normally distributed and therefore non-parametric methods were employed for all analyses. Continuous variables were described using the median and inter-quartile range. Differences in background characteristics of included (n = 58) and excluded (n = 35) participants were tested using Mann-Whitney U test for continuous variables and χ^2^-test for categorised variables.

Agreement between the accelerometer and the pedometer was assessed in several ways. Absolute step-count measurements were compared using the Wilcoxon Signed-Rank Test. The relative agreement between pedometer-derived step-counts and various accelerometer measures of PA was examined by determination of the Spearman rank correlation coefficient (ρ).

Total agreement between accelerometer-derived and pedometer-derived step-counts was evaluated by determination of the 95% limits of agreement. The difference between both measures of step counts was plotted against the mean of both measures. Since there was a statistically significant negative correlation between these variables, which was not resolved by transformation, the limits of agreement were estimated by a regression-based method [35]. To test whether the limits of agreement varied by baseline BMI (25.0-29.9 kg/m^2 ^vs. ≥30.0 kg/m^2^) or gestational age (11-14 vs. ≥20 weeks' gestation), interactions terms were added to regression models, and absolute residuals were compared by Student's t-tests.

The classification of participants according to whether or not they recorded a daily mean of at least 8,000 or 10,000 steps/day was compared between pedometer and accelerometer by calculating Cohen's kappa over 2 × 2 contingency tables. Kappa was also determined to assess the agreement between those reaching 8,000 pedometer steps/day and those achieving 30 mins MVPA measured by accelerometer. Kappa values 0.81-1.00 were regarded as almost perfect, 0.61-0.80 good, 0.41-0.60 moderate, 0.21-0.40 fair and 0.0-0.20 slight [36].

P-values and confidence intervals for ρ were estimated by bootstrapping over 5,000 iterations. P < 0.05 was considered statistically significant. The majority of statistical analyses were performed using SPSS 17.0 for Windows (SPSS Inc., IL), however bootstrapping methods used Stata 10.2 (StataCorp, TX).

## Results

Of the 93 women recruited, 32 (34%) had less than three valid days of accelerometer data, 17 (18%) had three and 44 (47%) had four days. All valid days from women with at least three valid days (n = 61) were included in further analyses, excluding three women who did not have pedometer data. The final study sample consisted of 58 women (62% of those recruited). The excluded women (n = 35) were younger (medians 28 vs. 32 years, p = 0.018), more often smokers during the previous year (46 vs. 13%, p = 0.002) and fewer of them were highly educated (5 vs. 59%, p < 0.001), but gestational age, BMI, parity, ethnicity, marital status and employment status were similar to those of the included women. The background characteristics of the included women are described in Table 1. The median age was 32 years and median BMI 29.3 kg/m^2 ^(range from 25.3 to 46.2 kg/m^2^).

**Table 1:** Background characteristics of the participants

	Participants
**Continuous variables, medians (interquartile range)^1^**	
Age, years	32 (27-36)
Weeks' gestation	13 (12-20)
Body mass index, kg/m^2^	29.3 (27.5-33.8)
**Categorized variables, numbers (%)**	
Weeks' gestation category	
11-14	32 (55.2)
≥20	26 (44.8)
BMI category	
25.0-29.9 kg/m^2^	35 (60.3)
≥30.0 kg/m^2^	22 (39.7)
Parity	
0	27 (46.6)
1	21 (36.2)
≥2	10 (17.2)
Education (highest qualification)^2^	
GCSE or equivalent (at age ≥16 years)	9 (17.0)
A level or equivalent (at age ≥18 years)	13 (24.5)
Degree or higher postgraduate qualification	31 (58.5)
Ethnicity	
White	48 (88.9)
Other	6 (11.1)
Smoked during the last year	
Yes	7 (12.7)
No	48 (87.3)
Employed at the beginning of pregnancy	
Yes	48 (84.2)
No	9 (15.8)
Hours of employment^3^	
Full time (≥37 h/week)	30 (63.8)
Part time (<37 h/week)	17 (36.2)
Living with a partner/husband	
Yes	55 (96.5)
No	2 (3.5)

^1 ^n = 58 for continuous variables

^2 ^GCSE: General Certificate of Secondary Education. Higher postgraduate qualification includes Professional Graduate Certificate in Education (or Postgraduate Certificate in Education), MSc, PhD etc.

^3 ^including 48 women who were employed

### Descriptive activity data

The median wear time of the accelerometer was 13 hours 40 min/day (Table 2). The women were sedentary for most of that time and total active time (median 4 hours 50 min/day) mainly comprised of light intensity activity. The median time spent in MVPA was 18 min/day.

**Table 2:** Descriptive data on physical activity measures among participants with at least 3 valid days (n = 58)^1^

	Median (interquartile range)	Range
**Accelerometer**		
Total included wearing time ^2^	821.8 (754.0-869.3)	608.0-1,111.0
Sedentary time ^2^	514.0 (464.3-583.1)	255.7-849.8
Total activity time ^2^	290.9 (245.8-340.1)	125.7-473-5
Light activity ^2^	271.3 (218.8-315.4)	99.3-429.3
Moderate activity	18.0 (11.4-29.1)	5.3-70.0
Vigorous activity	0.0 (0.0-0.0)	0.0-4.3
Moderate or vigorous activity	18.0 (11.7-30.1)	5.3-70.0
Total counts/day	202,680 (166,951-248,344)	92,131-429,497
Average counts/min	256.3 (209.5-323.7)	131.0-615.5
Step counts/day	5,687 (4,452-7,086)	1,545-11,453
**Pedometer**		
Step counts/day ^2^	5,961 (3,727-8,510)	267-12,833

^1 ^Min/day unless otherwise specified. The values represent the average of all valid days.

^2 ^A normally distributed variable

### Agreement between continuous pedometer and accelerometer measures of PA

**Figure 1:**
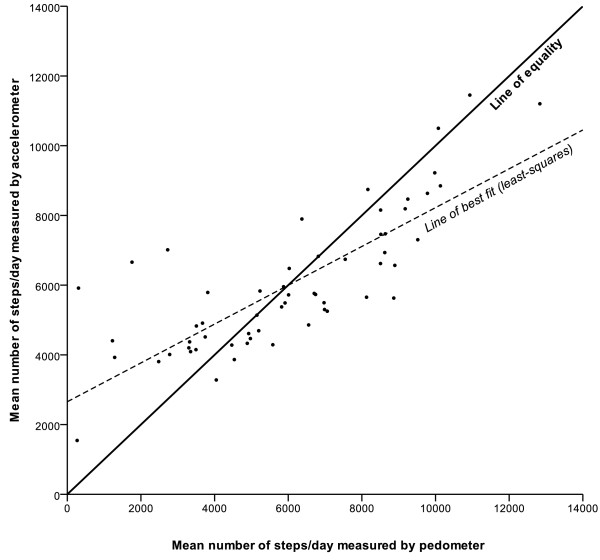
**Daily step counts measured by accelerometer and pedometer**. The figure also shows the line of equality and (least-squares) line of best fit (n = 58).

There was no significant difference between the overall step counts recorded by the pedometer and the accelerometer (medians 5961 vs. 5687 steps/day, p = 0.37, Table 2). Pedometer step counts were significantly correlated with all accelerometer measures of PA except for sedentary time (Table 3). The correlation was good for accelerometer step counts (r = 0.78) and moderate (r = 0.36 to 0.54) for all other measures of PA. Pedometer step counts are plotted against accelerometer step counts in Figure 1.

**Table 3:** Spearman correlation coefficients between pedometer step counts and accelerometer measures of physical activity (n = 58).

	Correlation coefficient	95% confidence intervals	p-value
Sedentary time (min/day)	-0.30	-0.51 to 0.05	0.023
Total activity time (min/day)	0.40	0.13 to 0.63	0.002
Light activity (min/day)	0.36	0.10 to 0.58	0.006
Moderate or vigorous activity (min/day)	0.47	0.18 to 0.69	<0.001
Total counts/day	0.51	0.24 to 0.72	<0.001
Average counts/min	0.54	0.28 to 0.74	<0.001
Step counts/day	0.78	0.59 to 0.90	<0.001

**Figure 2:**
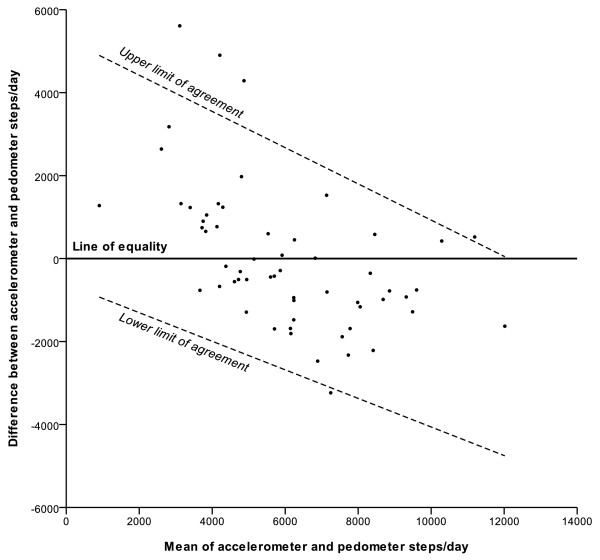
**Differences in step counts/day between accelerometer and pedometer plotted against the mean of both (n = 58)**. The limits of agreement were calculated using a regression method previously described by Bland & Altman [34]. The formula for the 95% limits of agreement is: [2339-0.391 × (mean of accelerometer and pedometer)] ± 2.46 × [1201-0.019 × (mean of accelerometer and pedometer)].

Despite these statistically significant correlations, the 95% limits of agreement were very broad, ranging between -2690 to 2656 steps/day for the mean value (mean of accelerometer and pedometer steps/day = 6026) (Figure 2). The limits of agreement also varied substantially over the range of values, indicating a differential bias. At the lowest recorded step count (mean of accelerometer and pedometer steps/day = 906), the limits were -927 to 4897 steps/day (a range of 5,824), indicating that the accelerometer was on average recording more steps/day than the pedometer. In contrast, at the highest step count value (mean of accelerometer and pedometer steps/day = 12,018) the limits were -4753 to 33 steps/day (a range of 4,786) indicating that while the level of random disagreement had decreased, the direction of bias had reversed, with the accelerometer recording less steps/day than the pedometer on average.

BMI and gestational age did not modify the limits of agreement as there were no statistically significant differences in the slope of the regression line (p = 0.28 for BMI, p = 0.68 for gestational age) or in the absolute spread from the regression line (p = 0.64 for BMI, p = 0.35 for gestational age).

### Agreement between categorised pedometer and accelerometer measures of PA

Based on the accelerometer data, 15 (26%) of these women recorded ≥30 mins MVPA/day, 12 (19%) recorded ≥8,000 steps/day and 3 (5%) recorded ≥10,000 steps/day. The pedometer data showed that 18 (29%) of women recorded ≥8,000 pedometer steps/day and 4 (7%) recorded ≥10,000 steps/day.

There was moderate agreement between those achieving ≥8,000 pedometer steps/day and those achieving ≥30 min MVPA/day (kappa = 0.45, 95% CI: 0.24 to 0.67) (Table 4). Agreement between the pedometer and the accelerometer in categorising women to <8,000 or ≥8,000 steps/day was good (kappa = 0.63, 95% CI: 0.43 to 0.83). Very few women achieved ≥10,000 steps/day with either of the monitors (N = 3 for accelerometer and N = 4 for pedometer), thus the agreement between these was artificially high (results not shown).

**Table 4:** Agreement between categorised pedometer and accelerometer measures of physical acticity, Kappa (95% confidence intervals).

**Accelerometer data**^**1**^	**Kappa (accelerometer data vs. ≥8,000 pedometer step counts/day)**^**2**^	95% confidence intervals
≥30 mins MVPA/day	0.45	0.24 to 0.67
≥8,000 steps/day	0.63	0.43 to 0.83

^1 ^15 (26%) of the participants recorded ≥30 mins MVPA/day and 12 (19%) recorded ≥8,000 steps/day

^2 ^18 (29%) of the participants recorded ≥8,000 pedometer steps/day

## Discussion

Large scale trials are needed to assess the potential impact of increased PA on reducing pregnancy complications and these trials should ideally use objective measurement methods to measure changes in PA [1,3,37]. Pedometers would be a cost-effective measurement tool for large studies, provided that the simple step count measure is broadly comparable to the more specific accelerometer data. Our results show that although there was a significant correlation between pedometer step counts and most accelerometer measures of PA and no difference in median step counts between the two devices, the 95% limits of agreement were very broad, especially among those participants who were less active. Additionally, the direction of difference between the monitors appeared to reverse across the range of activity levels suggesting a complicated pattern of disagreement. Agreement between the monitors in categorising participants as active and inactive varied from fair to good depending on the criteria adopted, being good when achievement of ≥8,000 steps/day was used as the criterion.

Pedometer step counts have been compared to accelerometer data in a number of previous reports [18,20-23], all of which used one of the Actigraph/CSA/Manufacturing Technology Inc. (MTI) accelerometer models and one of the Yamax pedometer models, as in the present study. These accelerometer models are not entirely comparable to each other in measuring steps and activity counts [38,39]. The study by Harrison et al. [18] included 30 overweight or obese pregnant women at 26 to 28 weeks' gestation in Australia. The participants wore an accelerometer (GT1M Actigraph) and a pedometer for 5 to 7 days and the accelerometer data processing rules were very similar to those used in our study. Despite a statistically significant correlation (r = 0.69, p < 0,01) between the step counts of each monitor, the mean difference was 505 steps/day and the limits of agreement were large (from -2491 to 3501 steps/day).

The other studies were not conducted in pregnant women and generally included subjects who were more active than our participants. However, the findings were essentially similar to those in the present study. Tudor-Locke et al. [20], in a study of 60 adult volunteers in South Carolina, USA, observed a high correlation between accelerometer (CSA model 7164) and pedometer step counts (r = 0.86), but the accelerometer detected 1845 ± 2116 more steps/day on average than the pedometer and the limits of agreement were even broader (-2387 to 6077 steps/day) than reported in the present study. In a larger study of older adults in the UK (n = 121) [23], Harris et al. reported that pedometer step counts were highly correlated to the accelerometer (Actigraph GT1M) step counts (r = 0.86) and the mean step counts were similar. However, the limits of agreement were again large, around -3500 to 3500 [23]. Ramirez-Marrero et al. [22] reported similar findings among 58 adults with HIV in Puerto Rico. Although the limits of agreement were not calculated in that study, individual variation in differences in step counts seemed to be large. When comparing pedometer step counts to other accelerometer (Actigraph model 7164) measures of PA, the correlations observed in the present study are generally similar, although weaker than in the previous studies [20,22,23]. Macfarlane et al. [21] observed among 57 adult volunteers in Hong Kong that the means for accelerometer (MTI model 7164) measures of PA increased with increasing pedometer step counts, but the confidence intervals were broad.

Amongst the previous studies, Tudor-Locke et al. [20] were the only authors to conclude that agreement between pedometer step counts and accelerometer measures of PA was unacceptably low, despite others also reporting broad limits of agreement [18,23] or confidence intervals [21]. Future studies should pay more attention to correct interpretation of Bland-Altman plots and limits of agreement.

The present study confirms the findings of these studies in a sample of 58 overweight and obese pregnant women. Although there are currently no methods available to calculate 95% confidence intervals for the limits of agreement determined by a regression based method, it is important to note that a larger sample size would not have affected the size of the limits of agreement. There are also no guidelines for acceptable 95% limits of agreement for step counts. We propose that they should be no larger than ± 500 steps/day (i.e. a range of 1000 steps/day), which is likely to correspond to a maximum of 10 min difference in the duration of MVPA, such as brisk walking [33], and may therefore be of clinical and public health importance.

The difference between the accelerometer and the pedometer step counts was correlated to the mean of both measures in the present study, but not in the others [18,20,23]. In this study, the difference between the step counts was in the opposite direction for less active and more active women, i.e. accelerometer detecting more steps among less active women and pedometer detecting more steps among more active women. This discrepancy may be related to the general limitations of the monitors or differences in their sensitivity to detect PA. Pedometer accuracy is reported to be diminished at slow walking speeds, especially below 3 miles/h [31,40] and both active and inactive participants undertook many episodes of light intensity activity in the present study. This may also be the case with some accelerometers, although the GT1M model used in our study has been shown to have lower intermonitor variability and lower sensitivity for light intensity activity than the preceding 7164 model [38,39]. On the other hand, the previous CSA model has also been reported to erroneously detect slightly more nonsteps e.g. when travelling by a motor vehicle [41].

The accuracy of the latest Actigraph accelerometer model (GT3X) and Yamax Digiwalker SW-200 was recently investigated in 30 pregnant women [19]. Both monitors underestimated the number of steps especially at slow walking speeds, but positioning the monitors at a tilt angle did not correlate with the percentage of actual steps detected by either monitor. In contrast, Crouter et al. [31] suggested that the tilt angle reduced the accuracy of spring-levered pedometers in overweight or obese adults. The tilt angle may also reduce the accuracy of accelerometers in assessing vertical movement, which may happen more often among overweight and obese than normal weight people [17]. The tilt angle was not directly measured in the present study. However, BMI and gestational age did not significantly modify the results of the Bland-Altman plot suggesting that the potential effect of the tilt angle on the results may have been the same regardless of BMI or gestational age.

We also assessed agreement between the monitors in categorising participants as active or inactive. Whilst agreement was relatively good (Kappa 0.63) when using 8,000 steps/day as the criterion for both monitors, agreement was lower (Kappa 0.45) when comparing participants achieving 8,000 pedometer steps/day to those achieving 30 min of MVPA/day measured by accelerometer. Of the previous studies, Ramirez-Marrero et al. [22] reported fair agreement between 10,000 pedometer steps/day and 150 min MVPA/week (Kappa 0.25, p = 0.01). These discrepancies between pedometer and accelerometer in categorising participants into active and inactive may partly be due to the data processing rules, such as selection of the epoch length and intensity cut points to define MVPA for the accelerometer data.

Accelerometry should not be regarded as a gold standard to measure free-living PA nor necessarily as a more accurate method of measuring daily steps than pedometer. Although the previous version of the Actigraph accelerometer the only commercially available accelerometer [28] that correlated reasonably with doubly labelled water, most validation studies have been conducted in controlled environments. Validity is lower when applied to free-living settings [17]. Two armband accelerometers have also recently been shown to be highly correlated with doubly labelled water in free-living conditions [42]. Both the accelerometer and the pedometer measure biomechanical body movement. Hence, validity of the monitors against energy expenditure should not be a major concern when assessing agreement between these devices.

This study had some limitations. Firstly, although the participants were asked to record the times when they wore the monitors, we cannot be sure that both monitors were worn exactly for the same time. Some women had very low pedometer step counts but moderate accelerometer step counts suggesting that the wearing time may have been different for each monitor or the pedometer may have been in a tilt angle. Therefore, studies in controlled conditions, such as by Connolly et al. [19], would be necessary to be certain that monitors were worn for exactly the same time. Secondly, almost all of our participants were in the first or second trimester of pregnancy and therefore we do not know whether the results can be generalized to the third trimester, when activity decreases and the abdominal circumference is much larger. On the other hand, these results may be generalized to non-pregnant overweight and obese women of similar age.

Thirdly, participation was low (33%) and 34% of the participants were excluded because of fewer than three valid days of accelerometer data. The activity levels of the participants were similar or slightly lower than those reported in pregnant women in other comparable studies [5,6,16,43]. The purpose of this study, however, was to compare methods of measurement, rather than obtain representative estimates of PA levels in pregnancy.

## Conclusions

Comparing median step counts or assessing correlation coefficients over-estimates agreement between pedometer and accelerometer data. Examination of the 95% limits of agreement revealed a substantial lack of agreement between step counts measured by the two types of monitor. Pedometer step counts were not comparable to accelerometer data at an individual level in overweight and obese pregnant women. The choice of measurement method may depend on the target of the intervention. For example, accelerometers may be better at assessing changes in PA in trials which promote increases in moderate or vigorous PA or reduction in sedentary time while spring-levered pedometers may be more appropriate for studies evaluating walking interventions in more active populations.

## Competing interests

The authors declare that they have no competing interests.

## Authors' contributions

TIK participated in the design of the study and statistical analyses and was responsible for writing the manuscript. PWGT participated in statistical analyses. CM recruited the participants and collected all data. LP participated in the design of the study. SCR and RB participated in the design of the study and supervising data collection and analyses. All authors participated in interpreting the results and revising the manuscript, and they read and approved the final manuscript.

## Pre-publication history

The pre-publication history for this paper can be accessed here:

http://www.biomedcentral.com/1471-2458/11/501/prepub
